# First Annual Announcement of the Medical Department of the Lind University, at Chicago, Ill., 1859–60

**Published:** 1859-06

**Authors:** 


### EDITORIAL.

### FIRST ANNUAL ANNOUNCEMENT OF THE MEDICAL DEPARTMENT OF LIND UNIVERSITY, AT CHICAGO, ILL., 1859-60.

As journalists, we feel bound to chronicle so important an event as the establishment of a new medical school in Chicago. As a teacher, we feel called upon to make some comments on the peculiar claims to superiority put forth in this announcement. As it is in the hands of all our subscribers, we are saved the room necessary to give the announcement entire, which we should otherwise prefer to do.

The following extracts will sufficiently indicate the changes proposed by this new organization.

Plan of Instruction.—Each college term will consist of two departments, essentially distinct from each other, but carried on simultaneously. The first, called the *junior department*, will embrace full courses of lectures and demonstrations on the following branches, viz: Descriptive Anatomy, Physiology and Histology, Materia Medica and General Therapeutics, General Pathology and Public Hygiene, Inorganic Chemistry, and Practical Anatomy, under the direction of the demonstrator, and is designed for all students attending the first course of lectures.

All medical students in this department will be examined at the end of the term, on the branches taught in the course, and if such examination be satisfactory, it will be final for those branches.

The second, called the *senior department*, will embrace full courses of lectures on the principles and practice of Surgery, Surgical Anatomy, Obstetrics and Diseases of Women and Children, Practice of Medicine, Organic Chemistry and Toxicology, Medical Jurisprudence, Clinical Medicine and Surgery in the Hospital, and Dissections under the demonstrator, and is designed for the students attending their second course.

The regular annual course of lectures in the medical department of the Lind University, will commence on the second Monday in October next, and end on the first Monday in March following.

The class in each department will receive *four* regular lectures daily, throughout the term.

By carefully noting this plan, it will be seen that it differs from that pursued in all the colleges in the United States, (except that at Ann Arbor, Mich.,) in that it proposes to make a full course of lectures comprise 430 lectures, delivered in twenty weeks, instead of 576 lectures, delivered in sixteen weeks, as is the present practice. The new faculty think that "the attempt to teach the whole of medical science and art, by six or seven professors, in four months," is a "defect" of a most "mischievous character," but that this can very readily be effected by five professors in five months, and by a smaller number of lectures. The simple statement of this change is sufficient to show that it is not an improvement.

2. "The last prominent defect in the prevailing system of medical instruction, which we shall mention, consists in requiring the student to attend so many lectures daily, that he has no adequate time for reflection," etc. So say the reformers. Is six hours a day too much time for healthy young men to be instructed, during our term of four or five months? Let us look at our public schools, and see what is required of boys twelve or fourteen years of age. Such boys are kept six hours in school, and required to get lessons as various and as numerous as those required of medical students. In the high school much more is required, and there is no good literary institution or college in which more application is not *required* for nine or ten months of the year than is *expected* of medical students for four or five. Six hours for lectures, and a suitable time for reading, is no more than is requisite for mental discipline, and for keeping students from indolent habits and dangerous associations in a city; and the assertion that it is too much, contains an error to which the student should by no means listen. Having had considerable experience, and some association with men now eminent in the profession, we think it safe to assert that twelve hours a day for study, for six years, is not too much for acquiring a proper knowledge of the science of medicine—none too much for the proper development of the mental faculties, and, properly divided, none too much for health.

3. The medical department of the Lind University proposes to reform and improve medical education, by providing that a student shall enter practice after attending one course of lectures on anatomy, comprising 80 lectures, instead of two courses of 96 each, as is now the custom in medical colleges. So of surgery and other branches. Is this reform? The argument that the study of certain branches must precede others, is fallacious. Anatomy and surgery should go hand in hand—so of the study of medicinal substances and their uses. In regard to this curtailing the number of courses, and of lectures on important branches, we feel inclined to quote from this circular, the language employed in regard to the courses of the first medical colleges of this country—"No parallel to this absurdity can be found in any other branch of education in this country."

The circular proceeds to say, that it was these defects that led to the organization of the American Medical Association, in 1846–7. If the end and object of this association was and is to reduce the number of lectures, without improving their quality—to lengthen the term without adding to the means of teaching or of learning—if the programme of the medical department of the Lind University be its culminating point—(which we are far from believing)—then indeed, has the "mountain labored and brought forth a mouse."

For ourselves, we regard this new plan as utterly visionary. We propose to adhere to the plan of instruction now generally pursued throughout the country; to improve upon and perfect, if practicable; but not to destroy it. We believe it to be essentially good, and adapted to the wants of the profession, and especially of students in this country. Students are not generally wealthy, and but few of them have the means of attending lectures more than four months. Even the eloquence of these professors has often failed to induce them to remain that length of time, to hear lectures for which they had already paid. It is a system by which almost every eminent and learned man in this country has been principally educated, and we conceive that denouncing it as the way "in which the country (and especially the western part of it) is kept full of half-educated physicians," is neither just nor wise—neither calculated to improve the profession, nor to assert its just claims to respect before the public. It is an unjust attack upon physicians and schools, especially in the West, which irregular practitioners will not fail to turn to account.

There is still another point in this announcement, which must not be passed over without notice. It states, "There will be two cliniques in the College, and four in the Mercy Hospital, each week." What is this Mercy Hospital, and how do these gentlemen acquire their rights to dispose of it?

We can answer this question. In 1851, some of the professors of Rush Medical College, and other persons, friends of the institution, established the "Illinois General Hospital," with a charter obtained from the legislature. This, after being in full operation for some months, was transferred to the present proprietors of the Mercy Hospital, or their purchasers, together with "all the furniture, beds, bedding, stoves, chairs, tables, carpeting, and other fixtures belonging to, and hitherto in the use and possession of said Illinois General Hospital of the Lakes."

"And said party of the second part covenants and agrees to and with said parties of the first part, in consideration of said sale and transfer, that the trustees of the Rush Medical College, of said City of Chicago, shall, at all times hereafter, so long as said Mercy Hospital shall be maintained," * * * * "be permitted to appoint attending physicians and surgeons for the wards of said Mercy Hospital." The right was reserved to the proprietors of the Mercy Hospital, "to appoint one attending physician, whose term of office shall be during the vacation of Rush Medical College."

The members of the faculty of the medical department of the Lind University, who propose to give clinics in this hospital, during the coming winter, hold their position in violation of the above agreement, and it will be interesting to discover by what kind of ethics, men so conscientiously opposed to a lecture term of four months, that they could no longer continue to participate in it, will be able to retain places, and the use of property, to which they have no right. Their course in reference to this point will no doubt be such as high-minded men always pursue. It would be unjust to suspect them of any design, which, if carried into effect, would be derogatory to their characters as honorable men, and we therefore defer comment until their decision shall be made known.

"The object of the medical faculty of this university is to establish a medical school on such a basis as will afford facilities for as thorough a medical education as can be obtained in Europe." It will be admitted by all, that the object is still sufficiently far from its accomplishment. In a city which has no hospital, excepting one established by Rush Medical College, and in which the united efforts of the profession have as yet been insufficient to induce the authorities to open one, these gentlemen create a division, by which the influence of the medical faculty is likely to be diminished. Where there is scarcely a rudiment of a medical library, or a museum, they propose to divide instead of concentrating efforts to provide them. The tendency of this movement is essentially destructive. It proposes to add nothing whatever to the education of the student, the requirements for graduation, or the means of acquiring knowledge. In many respects, it diminishes these; and whatever other merits it may have, seems to us very far from having that of improvement or reform.

