# Correction

**Published:** 1859-07

**Authors:** 


### CORRECTION.

In speaking of the number of lectures to comprise a course in the Lind University, 430 was printed instead of 480 as written. The number embraced in a course of sixteen weeks was correctly stated at 576.

We have been informed that in the article referred to, injustice has been done to the University of Michigan in classing it with the Lind University. It appears that the course in the Michigan University embraces the full number of lectures, although given in a longer term. We cheerfully make this correction.

